# Exploring cell membrane water exchange in aquaporin-4-deficient ischemic mouse brain using diffusion-weighted MRI

**DOI:** 10.1186/s41747-021-00244-y

**Published:** 2021-10-07

**Authors:** Takuya Urushihata, Hiroyuki Takuwa, Manami Takahashi, Jeff Kershaw, Yasuhiko Tachibana, Nobuhiro Nitta, Sayaka Shibata, Masato Yasui, Makoto Higuchi, Takayuki Obata

**Affiliations:** 1 Department of Functional Brain Imaging Research, Institute for Quantum Medical Science, QST, Chiba, 263-8555 Japan; 2 Applied MRI Research, Department of Molecular Imaging and Theranostics, Institute for Quantum Medical Science, QST, Chiba, 263-8555 Japan; 3 Keio Advanced Research Center for Water Biology and Medicine, Keio University, Tokyo, 160-0016 Japan

**Keywords:** Aquaporin 4, Cell membrane, Cerebral infarction, Diffusion magnetic resonance imaging, Experimental animal models

## Abstract

**Background:**

Aquaporin-4 is a membrane channel protein that is highly expressed in brain astrocytes and facilitates the transport of water molecules. It has been suggested that suppression of aquaporin-4 function may be an effective treatment for reducing cellular edema after cerebral infarction. It is therefore important to develop clinically applicable measurement systems to evaluate and better understand the effects of aquaporin-4 suppression on the living body.

**Methods:**

Animal models of focal cerebral ischemia were created by surgically occluding the middle cerebral artery of wild-type and aquaporin-4 knockout mice, after which multi-*b*-value multi-diffusion-time diffusion-weighted imaging measurements were performed. Data were analyzed with both the apparent diffusion coefficient (ADC) model and a compartmental water-exchange model.

**Results:**

ADCs were estimated for five different *b* value ranges. The ADC of aquaporin-4 knockout mice in the contralateral region was significantly higher than that of wild-type mice for each range. In contrast, aquaporin-4 knockout mice had significantly lower ADC than wild-type mice in ischemic tissue for each b-value range. Genotype-dependent differences in the ADC were particularly significant for the lowest ranges in normal tissue and for the highest ranges in ischemic tissue. The ADCs measured at different diffusion times were significantly different for both genotypes. Fitting of the water-exchange model to the ischemic region data found that the water-exchange time in aquaporin-4 knockout mice was approximately 2.5 times longer than that in wild-type mice.

**Conclusions:**

Multi-*b*-value multi-diffusion-time diffusion-weighted imaging may be useful for *in vivo* research and clinical diagnosis of aquaporin-4-related diseases.

**Supplementary Information:**

The online version contains supplementary material available at 10.1186/s41747-021-00244-y.

## Key points

- We investigated aquaporin-4 loss and focal brain ischemia with diffusion-weighted magnetic resonance imaging in a mouse animal model.
- Cell membrane water exchange time parameters were estimated by model fitting.
- The effect of aquaporin-4 loss is opposite in healthy and ischemic tissue.
- Diffusion-weighted magnetic resonance imaging may be useful for research and clinical diagnosis of aquaporin-4-related diseases.

## Background

Aquaporin-4 is a membrane channel protein that allows water molecules to be passively transported, is the most commonly expressed aquaporin on the feet of astrocytes in the mammalian brain [1, 2], and plays an important function in water movement across the blood-brain barrier [3–6]. It has also been reported that aquaporin-4 is involved in brain diseases such as ischemic stroke [7, 8], hydrocephalus [9, 10], Alzheimer’s disease [11–13], amyotrophic lateral sclerosis [14], traumatic brain injury [15], and epilepsy [16, 17]. These findings have led to the design of treatment strategies that target aquaporin-4 [18, 19].

Cytotoxic edema, a condition where increased water in brain cells causes swelling, is frequently observed in a variety of brain diseases [7, 20]. Unfortunately, the molecular and cellular mechanisms underlying the formation and resolution of edema are not yet fully understood, and there is still no clear treatment [20, 21]. In studies using animal models, aquaporin-4 expression is sharply increased in ischemic brain edema [22, 23], and aquaporin-4 knockout or inhibitor administration has been shown to be effective in reducing cellular edema [8, 24–26]. Aquaporin-4 inhibition has therefore been proposed as a treatment for cytotoxic edema. On the other hand, inhibition of aquaporin-4 is also known to cause astrocyte dysfunction, preventing recovery from ischemia [27–29]. The complex effects of aquaporin-4 on the formation of cytotoxic edema have not been fully evaluated, nor has a clear method for *in vivo* evaluation been established. A clinically applicable method that can reliably evaluate changes in cytotoxic edema caused by aquaporin-4 inhibition is needed for the future development of suitable drugs and other therapies.

Diffusion-weighted imaging (DWI) has been used as an important tool for the diagnosis of diseases such as stroke and cancer [30]. A quantity that is often estimated in DWI studies is the apparent diffusion coefficient (ADC). The ADC is said to be “apparent” because the complexity of *in vivo* tissue microstructure dictates that it is an indicator of the overall signal behavior from multiple diffusion-related processes, rather than the diffusion coefficient of a single water compartment. In fact, different ADCs may be attributed to different parts of an organism depending on the nature of diffusion in that component [31]. For example, as intracellular water diffusion is strongly obstructed by the cell membrane and many other structures inside the cell, it usually corresponds to a restricted diffusion component. On the other hand, diffusion through the extracellular space is comparatively, but not completely, free so it contributes to a hindered diffusion component. In addition, the disordered arrangement of the microvasculature implies that blood-water molecules display a diffusion-like behavior called intravoxel incoherent motion (IVIM). Accordingly, blood signal is attributed to an IVIM pseudo-diffusion component [32]. In general, the signals from each of these components contribute to the overall signal at low *b* values. However, the contribution from the IVIM component is negligible for *b* values above 500 s/mm^2^, and the signal from the hindered diffusion component is relatively small for *b* values greater than 2,000 s/mm^2^, which leaves the restricted diffusion component near the cell as the dominant contributor at high *b* values [31, 33]. Therefore, observing the signal over a wide range of *b* values may help to isolate the contributions of different diffusion components, in particular the restricted diffusion component, which is the component most likely to reflect the effects of aquaporin-4 on water transport.

In a previous study, we proposed and applied a DWI-based technique to quantitatively evaluate cell membrane water permeability for *in vitro* monoclonal cell suspensions [34]. The technique is based on a modification of the Andrasko-Kärger model [35–37], which is a simple two-compartment model with inter-compartmental compound exchange. Briefly, since the effect of membrane permeability on DWI measurements is highly dependent on diffusion-time [38, 39], the water exchange-time between compartments was estimated from differences in DWI signal attenuation at different diffusion-times. It was shown that the technique can be used to characterize differences in water exchange between aquaporin-4-expressing and non-expressing cells, and the results were consistent with data measured by coherent anti-Stokes Raman scattering microscopy [40]. This noninvasive DWI-based method may also be useful for the evaluation of cell membrane water permeability changes caused by aquaporin-4 abnormalities, and for the development of medicines targeting aquaporin-4.

In this study, we performed DWI on wild-type and aquaporin-4 knockout mice to evaluate the effects of aquaporin-4 suppression *in vivo*. Animal models with focal cerebral ischemia were surgically created by middle cerebral artery occlusion (MCAO), and multi-*b*-value multi-diffusion-time (MbMTd) DWI measurements were performed. ADCs were estimated and compared between the wild-type and aquaporin-4 knockout mice for both the ischemic and contralateral regions. The ischemic data was also analyzed with the two-compartment exchange model to quantitatively compare the water-exchange time and the other model parameters between mice with and without aquaporin-4.

## Methods

All experiments were performed in accordance with the institutional guidelines on humane care and use of laboratory animals and were approved by the Institutional Committee for Animal Experimentation of the National Institutes for Quantum and Radiological Science and Technology (QST). The datasets analyzed during the current study are available from the corresponding author on reasonable request.

### Animal preparation

A total of 6 C57BL/6J wild-type mice (both male and female, 20–30 g, 8–10 weeks; Japan SLC, Hamamatsu, Japan), and 7 aquaporin-4 knockout mice (both male and female, 20–30 g, 8–10 weeks) generated as described previously [41, 42] (acc. no. CDB0758 K: http://www.cdb.riken.jp/arg/mutant%20mice%20list.html), were used in the magnetic resonance imaging (MRI) experiments. All mice were housed individually in separate cages with water and food ad libitum. Mouse cages were kept at a temperature of 25 °C in a 12-h light/dark cycle. Overall, no clear differences in body weight and size were observed for any of the mice. MCAO was performed for all animals using the Tamura method [43], where a permanent occlusion is made at the proximal branch of the MCA in the left cerebral cortex. In this animal model, ischemia in the MCA region occurs soon after MCAO, and the infarction expands and peaks at 24 hours after surgery [43–46].

### MRI measurements

MRI measurements were performed at 2 h after MCAO surgery, which is during the initial stage of cytotoxic edema formation due to ischemia. This time window was selected because it was anticipated that the presence or absence of aquaporin-4 would contribute to differences in ischemic-related water movement during edema formation and before blood-brain barrier breakdown [3–6, 47]. All measurements were performed with a 7-T animal MRI (Kobelco and Bruker, Tokyo, Japan). The mice were initially anesthetized with 3.0% isoflurane (Escain, Mylan Japan, Tokyo, Japan), and then with 1.5% to 2.0% isoflurane and a 1:5 oxygen/room-air mixture during the MRI experiments. Rectal temperature was continuously monitored with an optical fiber thermometer (FOT-M, FISO, Quebec, QC, Canada), and maintained at 37.0 ± 0.5 °C (range) using a heating pad (Temperature control unit, Rapid Biomedical, Rimpar, Germany). Warm air was provided with a homemade automatic heating system regulated by an electric temperature controller (E5CN, Omron, Kyoto, Japan) throughout all experiments. During MRI scanning, the mice lay in a prone position on a MRI-safe cradle and were held in place with handmade ear bars.

MbMTd DWI was obtained using a pulsed-gradient spin-echo sequence with four-shot echo-planar acquisition (repetition time 3 s, echo time 115 ms, matrix size 128 × 128, spatial resolution 0.02 × 0.02 mm^2^, slice thickness 1.5 mm, gradient directions 3). The separation of the diffusion-gradient lobes (Δ) was set at 40, 70, and 100 ms to vary diffusion-time while keeping echo time constant. The diffusion-gradient duration (δ) was fixed at 7 ms for all experiments. For the pulsed-gradient spin-echo sequence, Δ-δ/3 is usually taken to represent the diffusion-time. For each Δ, the *b* value was increased from 0 to 8,000 s/mm^2^ in 11 steps (0, 2, 500, 1,000, 2,000, 3,000, 4,000, 5,000, 6,000, 7,000, and 8,000 s/mm^2^) by increasing the gradient amplitude. The multi-*b*-value DWI scan time for each Δ was about 6 min, which means that it took about 18 min for one set of MbMTd DWI. To check scan stability, 4 sets of MbMTd DWI were acquired for each animal in the study.

### DWI data processing

DWI data analysis was performed in MATLAB, version R2019a (MathWorks, Natick, MA, USA). Regions-of-interest (ROIs) were drawn in the ischemic and contralateral regions on T2-weighted images. The DWI data were averaged over the three gradient directions, and ADC maps were created by fitting to the logarithmic signals with respect to b-value using ordinary least squares. ADCs were estimated for five different *b* value ranges: 0–2,000 s/mm^2^, which is used in general clinical practice; 500–2,000 s/mm^2^, where it is thought the effect of the IVIM component is suppressed; and 2,000–4,000, 4,000–6,000, and 6,000–8,000 s/mm^2^, which are ranges where the restricted diffusion component is expected to be dominant. The average value of the ADC was calculated for each ROI and then averaged over animals. The signal-to-noise ratio (SNR) of the data was estimated using the air signal method [48],

Eq. 1 $$ \mathrm{SNR}={\left(\frac{\pi }{2}\right)}^{\frac{1}{2}}\times \frac{S_p}{S_{air}}, $$

where *S*_p_ is the signal in the ROI and *S*_air_ is the signal from a background region.

Evaluation of cell membrane water exchange-time was performed using a two-compartmental model with inter-compartment exchange (Fig. S1) [34]. Briefly, the model assumes that Δ is sufficiently long that the diffusion coefficient in the extracellular space (*D*_ex_) is approximately constant, while the diffusion coefficient in the intracellular space (*D*_in_) is inversely proportional to the diffusion time. The data was then analyzed using a constant *D*_ex_, and *D*_in_ was dependent on diffusion-time as below,

Eq. 2 $$ {D}_{in}=\frac{\alpha }{{\left(\varDelta -\delta /3\right)}^{\beta }}. $$

In this equation, α has dimensions of length squared in the case that β = 1, and β is a parameter inserted to test the assumption that *D*_in_ is inversely proportional to Δ-δ/3. Using the modified model, the DWI-based estimate of the exchange-time (τ_MRI_) is,

Eq. 3 $$ {\tau}_{\mathrm{MRI}}={F}_{ex}\cdotp {t}_{\mathrm{in}}={F}_{\mathrm{in}}\times {t}_{ex}, $$

where *F*_ex_ and *F*_in_ are the signal fractions of the extracellular and intracellular spaces, respectively, and *t*_ex_ and *t*_in_ are the lifetimes in the extracellular and intracellular spaces, respectively. Since it was demonstrated that β is close to 1 in a previous study [34], β was set to 1 in this study. The parameters *t*_in_, *F*_in_, *D*_ex_, and α were used as the free parameters while fitting to the model. *F*_ex_, *t*_ex_, and τ_MRI_ were then obtained using Eq. 3 and the constraint *F*_ex_ + *F*_in_ = 1.

### Statistical analysis

**Fig. 1:**
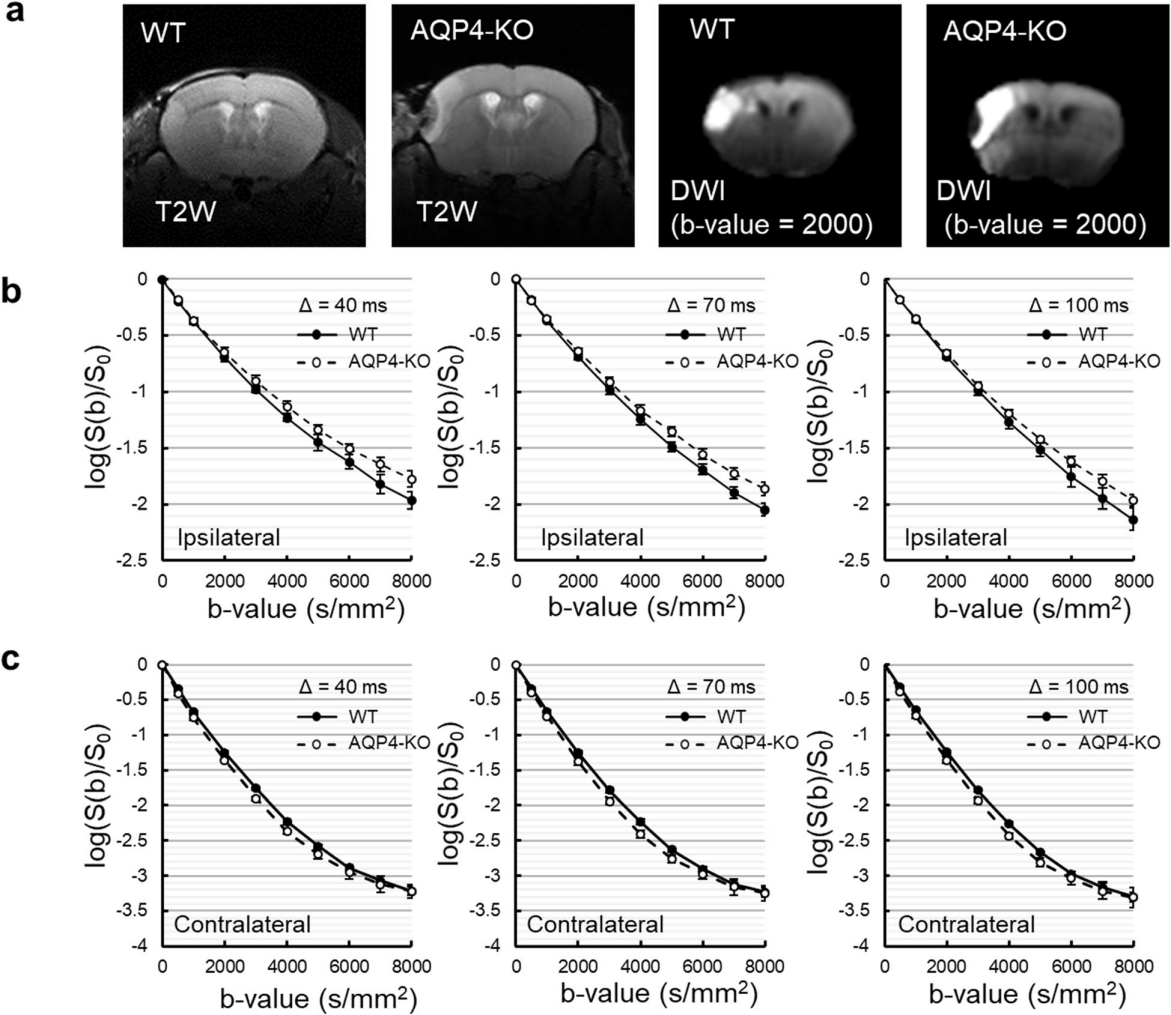
Multi-*b* value multi-diffusion-time images of wild-type (WT) and aquaporin-4 knockout (AQP4-KO) mice. Typical T2-weighted and diffusion-weighted (*b* value = 2,000 s/mm^2^) images for WT and AQP4-KO mice (**a**). The left side of the brain had a middle cerebral artery occlusion. Normalized *b*-value dependent signal decay for the ipsilateral (**b**) and contralateral (**c**) sides at different diffusion-times (Δ = 40, 70, and 100 ms). Solid lines indicate WT mice (closed circles with standard deviation bars) and broken lines indicate AQP4-KO mice (white circles with standard deviation bars)

**Fig. 2:**
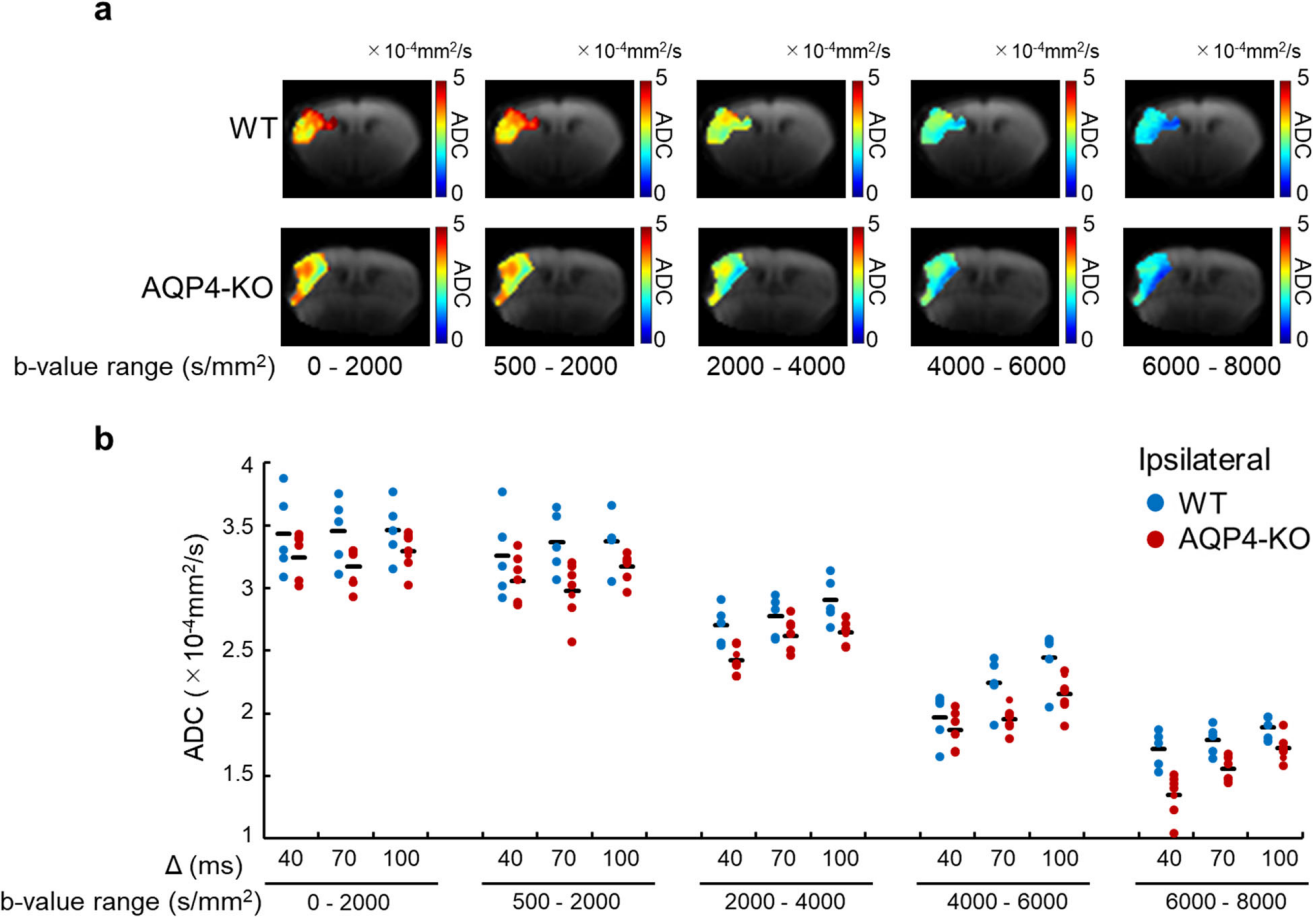
Apparent diffusion coefficient (ADC) in the ischemic region. Typical ADC maps of wild-type (WT) (top) and aquaporin-4 knockout (AQP4-KO) mice (top and bottom, respectively) focused on the ipsilateral region at Δ = 70 ms (**a**). The ADC in the ischemic region has a range of 0–5 × 10^−4^ mm^2^/s. The plots show the mean ADC of each WT (blue) and AQP4-KO (red) mouse for each *b*-value range and Δ (**b**). The black bars correspond to the mean over animals. The results of the statistical analysis are shown in Table 1

**Fig. 3:**
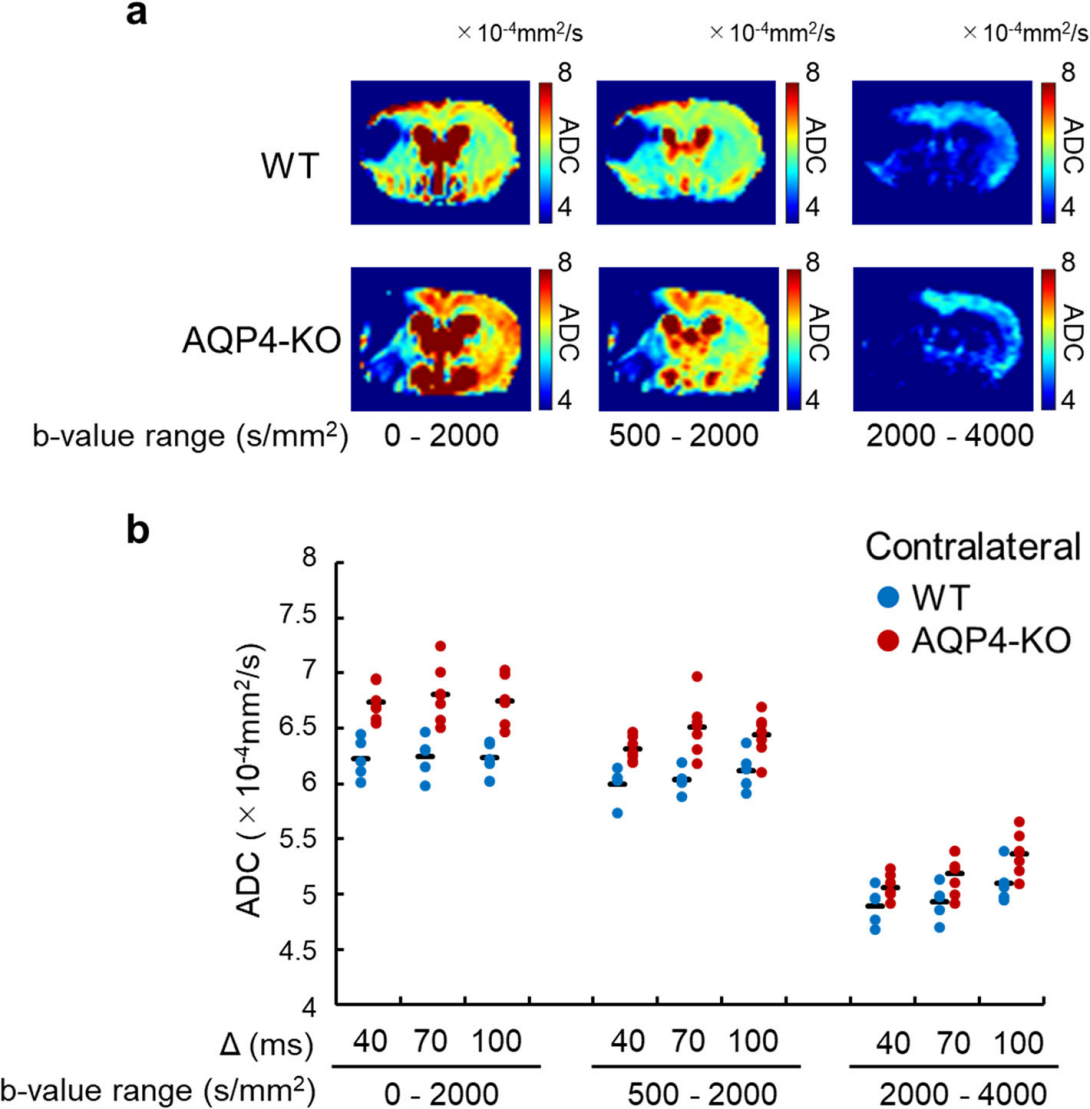
Apparent diffusion coefficient (ADC) in the contralateral region. Typical ADC maps of wild-type (WT) and aquaporin-4 knockout (AQP4-KO) mice (top and bottom, respectively), focused on the contralateral region (**a**). The ADC in the contralateral region has a range of 4–8 × 10^−4^ mm^2^/s. The plots show the mean ADC of each WT (blue) and AQP4-KO (red) mouse for each *b*-value range and Δ (**b**). The black bars correspond to the mean over animals. The results of the statistical analysis are shown in Table 2. The ADC maps and bar graphs for *b* = 4,000–6,000, and 6,000–8,000 s/mm^2^ are excluded because of low signal-to-noise ratio (see Fig. S3)

**Fig. 4:**
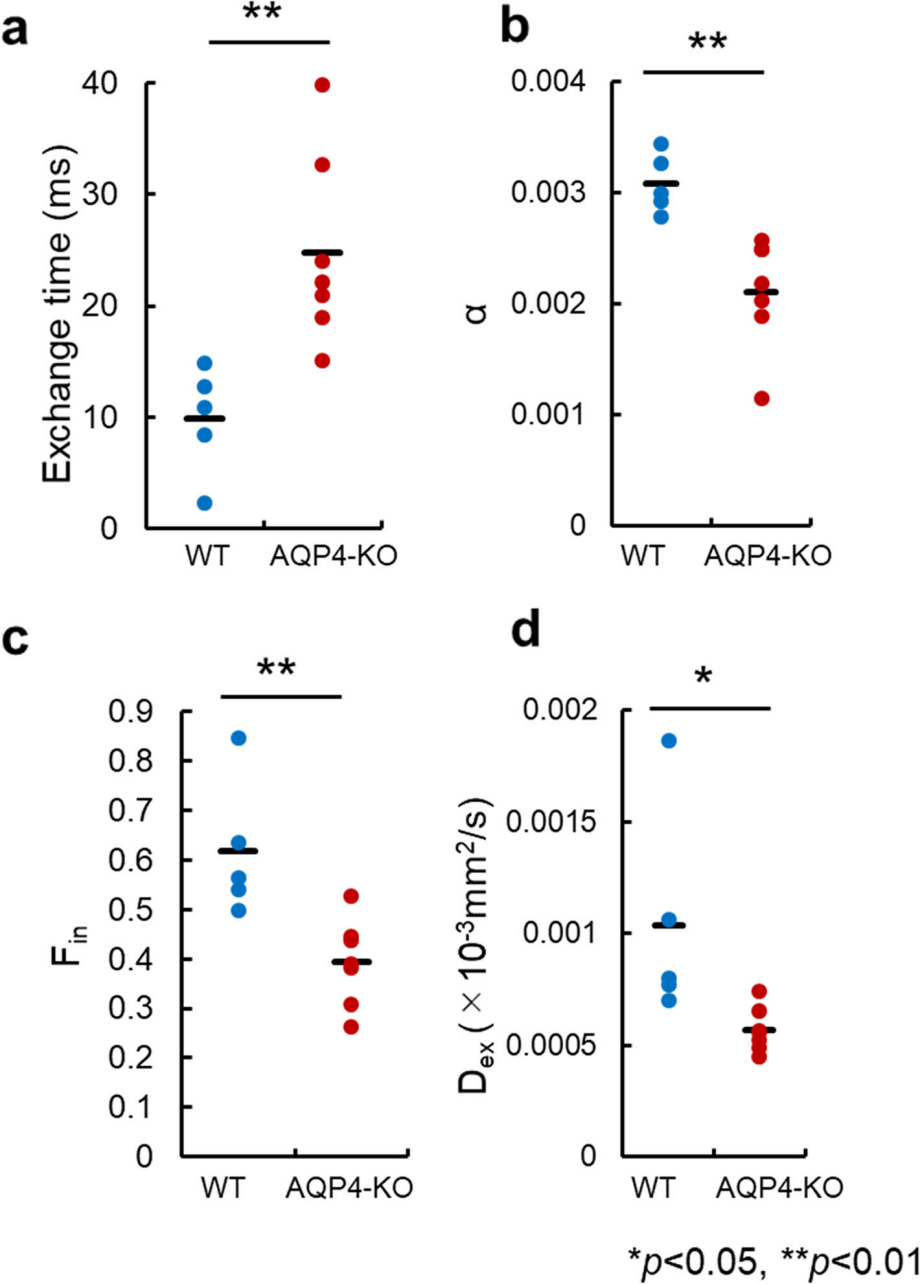
Estimates of the exchange model parameters in the ischemic region. The exchange model parameters were estimated by fitting to the diffusion-weighted imaging data in the ischemic region. Exchange-time (**a**), water exchange time between the intracellular and extracellular space; α (**b**), a parameter reflecting the mean cell volume; F_in_ (**c**), signal fraction from intracellular space. D_ex_ (**d**), apparent diffusion coefficient for the extracellular space. The plots show the mean for each wild-type (WT) (blue) and aquaporin-4 knockout (AQP4-KO) (red) mouse, while the black bars correspond to the mean over animals. * and ** indicate significant differences in the estimates from the WT and AQP4-KO mice (**a**, *p* = 0.006; **b**, *p* = 0.003; **c**, *p* = 0.004; **d**, *p* = 0.044; unpaired *t* test)

Statistical analyses were performed with the Statistics and Machine Learning Toolbox of MATLAB, version R2019a (MathWorks, Natick, MA, USA). Normalized DWI signals are presented as mean ± standard deviation over animals (Fig. 1). The ADCs and exchange model parameters are plotted for each animal with a bar indicating the mean over animals (Figs. 2, 3, and 4). Two-way analysis of variance (ANOVA), with mouse genotype and Δ as independent variables, was performed for the ADC estimated for each *b* value range. In addition, Student's *t* test was used to compare the exchange-model parameters between mouse genotype. The normality of each data set was confirmed with a Kolmogorov-Smirnov test. A *p* value < 0.05 was interpreted as being significant.

## Results

### MbMTd DWI signal changes caused by aquaporin-4 suppression and ischemia

As demonstrated by the high area on T2-weighted and diffusion-weighted images, MCAO produced a focal ischemic region on the left side of the mouse brain for both the wild-type and aquaporin-4 knockout mice (Fig. 1a). ROI-based analysis was performed on the ischemic (Fig. 1b) and the contralateral (Fig. 1c) regions of the MbMTd DWI, and the normalized signal was plotted against *b* value for all three values of Δ (40, 70, and 100 ms). In the ischemic region, the aquaporin-4 knockout mice had less *b* value dependent signal attenuation than the wild-type mice for all Δs (Fig. 1b). On the other hand, in the contralateral region, the aquaporin-4 knockout mice had greater *b* value dependent signal attenuation than the wild-type mice for all Δs (Fig. 1c). Longer Δ corresponded to greater signal attenuation for both mouse types and both regions (Fig. S2). The SNR in the contralateral region was less than 5 for *b* values above 5,000 s/mm^2^, so that data was excluded from subsequent analysis of that region (Fig. S3).

### Apparent diffusion coefficient

To characterize diffusion in the ischemic regions of both mouse types, ADCs were calculated for five *b* value patterns. Representative animal ADC maps are shown in Fig. 2a. For all *b* value ranges, the ADCs were significantly lower for the aquaporin-4 knockout mice than for the wild-type mice (Table 1). Moreover, the ADCs for the 2,000–4,000, 4,000–6,000, and 6,000–8,000 s/mm^2^ *b* value ranges showed a significant Δ-dependent difference (Fig. 2b, Table 1). The ADC in the ischemic region showed no significant interaction between mouse genotype and Δ for all *b* value ranges.

**Table 1:** Two-way ANOVA results (*F* values and *p* values) for the ADC on the ipsilateral side

*b* value (s/mm^2^)	0–2,000	500–2,000	2,000–4,000	4,000–6,000	6,000–8,000
Genotype	8.66 (0.006)^a^	12.4 (0.001)^a^	24.9 (< 0.001)^a^	15.4 (< 0.001)^a^	37.9 (< 0.001)^a^
Δ	0.244 (0.785)	0.938 (0.402)	9.92 (0.003)^a^	15.1 (< 0.001)^a^	14.7 (< 0.001)^a^
Genotype × Δ	0.241 (0.787)	0.675 (0.531)	0.684 (0.512)	1.17 (0.323)	2.08 (0.142)

The numbers within parentheses are *p* values, and those less than 0.05 were interpreted as being statistically significant (^a^). *ADC* Apparent diffusion coefficient, *ANOVA* Analysis of variance

As the SNR was very low above *b* = 5,000 s/mm^2^ (Fig. S3), the ADC in the contralateral region was calculated for only three of the *b* value ranges (0–2,000, 500–2,000, and 2,000–4,000 s/mm^2^). Representative ADC maps are presented in Fig. 3a. In contrast to the ischemic region, the aquaporin-4 knockout mice showed significantly higher ADC than the wild-type mice for each of the *b* value patterns (Fig. 3b, Table 2). Only the ADC for the 2,000–4,000 s/mm^2^ *b* value range showed a Δ-dependent difference. The ADC for all three *b* value ranges showed no significant interaction between mouse genotype and Δ (Fig. 3b, Table 2).

**Table 2:** Two-way ANOVA results (*F* values and *p* values) for the ADC on the contralateral side

*b* value (s/mm^2^)	0–2,000	500–2,000	2,000–4,000
Genotype	64.5 (< 0.001)^a^	38.85 (< 0.001)^a^	16.4 (< 0.001)^a^
Δ	0.171 (0.844)	1.896 (0.168)	6.86 (0.004)^a^
Genotype × Δ	0.0849 (0.919)	0.811 (0.454)	0.309 (0.737)

The numbers within parentheses are *p* values, and those less than 0.05 were interpreted as being statistically significant (^a^). *ADC* Apparent diffusion coefficient, *ANOVA* Analysis of variance

### Parameters of the compartmental exchange model

Estimates of the four parameters (τ_MRI_, α, *F*_in_, and *D*_ex_) obtained by fitting the exchange model to the ischemic data are shown in Fig. 4. The aquaporin-4 knockout mice showed a significantly longer water exchange-time than the wild-type mice (*p* < 0.006). The other parameters, α, *F*_in_, and *D*_ex_, had larger values for the wild-type mice (Fig. 4b–d), with the difference being significant (α, *p* < 0.003; *F*_in_, *p* < 0.006; *D*_ex_, *p* < 0.044).

## Discussion

In this study, we measured MbMTd DWI and compared ADCs estimated for ischemic and normal tissue of aquaporin-4 knockout and wild-type mice. In the ischemic region, aquaporin-4 knockout mice showed lower ADCs compared to wild-type mice, whereas higher ADCs were observed in the contralateral region. In addition, we compared parameter estimates obtained by fitting a two-compartment exchange model to the DWI signal. The cell membrane water exchange-time was approximately 2.5 times longer for the aquaporin-4 knockout mice than for the wild-type mice.

The observation of larger ADCs in normal tissue for the aquaporin-4 knockout mice is consistent with a report of an increase in ADC after the administration of the aquaporin-4 inhibitor TGN-020 [49]. It has also been reported that the volume of the extracellular space is comparatively large in aquaporin-4 knockout mice [50], and the suppression of aquaporin-4 expression by siRNAs in cultured rat cells reduces the size and total number of astrocytes [51]. As the contribution of the hindered diffusion component is relatively small for *b* values over 2,000 s/mm^2^, our observation of a larger difference between the contralateral ADCs of the wild-type and aquaporin-4 knockout mice for the two sub-2,000 s/mm^2^ *b* value ranges than for the 2,000–4,000 s/mm^2^ *b* value range (Fig. 3b, Table 2) may therefore reflect differences in the extracellular volume fraction between wild-type and aquaporin-4 knockout mice. Furthermore, as aquaporin-4 is expressed normally in wild-type mice, it might be hypothesized that the ADC of the restricted diffusion component in normal tissue is larger than it is for APQ4-KO mice. Unfortunately, we were unable to confirm this due to the low SNR of the 4,000–6,000 and 6,000–8,000 s/mm^2^ data on the contralateral side, but the trend of a decreasing difference between the ADCs of the two mouse genotypes with increasing *b* value range is consistent with the hypothesis (Fig. 3b, Table 2).

In ischemic tissue, the ADCs of the aquaporin-4 knockout mice were smaller than those of the wild-type animals for all *b* value ranges (Fig. 2b). Moreover, the gap between the ADCs of the two genotypes tended to become more significant for the higher *b* value ranges (Table 1). Ischemia-induced swelling of astrocytes and neuronal dendrites is expected to increase the volume fraction of the restricted diffusion component regardless of genotype [52, 53], in which case it is likely that the contribution of that component to the overall signal has greater weighting across all *b* value ranges. Therefore, it is possible that our observations in ischemic tissue reflect differences in the ADC and cell membrane water exchange-time of the restricted diffusion compartment for the two mouse types.

As the DWI signal can be affected by cerebral blood flow when using low *b* values [32, 54, 55], we compared the ADCs estimated using the 0–2,000 and 500–2,000 s/mm^2^ *b* value ranges. The ADCs were similar for the two ranges, indicating that differences dependent on mouse type were not related to the effect of cerebral blood flow on DWI.

The exchange model parameters in the living mouse brain were estimated by fitting to the MbMTd DWI data in the ischemia region (Fig. 4). Exchange time was about 2.5 times and significantly longer for the aquaporin-4 knockout mice than for the wild-type mice (Fig. 4a), which is consistent with previous *in vitro* studies [34, 40]. In addition, both the intracellular fraction, *F*_*i*n_, and the extracellular diffusion coefficient, *D*_ex_, estimates for the wild-type mice were greater than those for the APQ4-KO mice (Fig. 4c, d). This result for *D*_ex_ is a little unexpected as it could be argued that the smaller extracellular fraction, *F*_ex_ = 1 - *F*_in_, of the wild-type mice would result in a smaller value for the extracellular diffusion coefficient [56]. However, it is also possible that *D*_ex_ is larger for wild-type mice due to the need to break hydrogen bonds in clusters of water molecules so that a single molecule can pass through an aquaporin-4 channel [57–59]. Our results suggest that cell membrane water exchange abnormalities caused by aquaporin-4 loss might be detected in living animals.

A previous *in vitro* study found that signal attenuation for aquaporin-4 non-expressed cells at high *b* values (≈ 4,000–8,000 s/mm^2^) consistently decreased with increasing diffusion-time [34], which is similar to the behavior expected for a system where restricted diffusion dominates [60]. In the same *in vitro* study, attenuation of the DWI signal for aquaporin-4-expressed cells did not show a consistent trend with respect to increasing diffusion-time, from which it was suggested that the permeability of the cell membrane may have a significant effect on the signal at high *b* values. In contrast to those *in vitro* results, it was observed for the *in vivo* experiments performed here that the signal attenuation in ischemic tissue at high *b* values increased with increasing diffusion-time for both genotypes (Figs. 1 and S2). A similar observation was made in human subjects with stroke lesions [38].

The *in vivo* measurements of water exchange-time made for this work are shorter than those made on cultured cells using the same technique [34]. The reason for this result is not yet clear, but several factors may be involved. First, it may simply be that the water exchange-time of *in vivo* mouse cells is shorter than that of cultured cells regardless of aquaporin-4 expression. Also, it has been reported that acid pH increases the water permeability of aquaporin [61, 62]. It is therefore possible that the higher acidity of ischemic tissue increased the water permeability of aquaporin and hence shortened the water exchange-time [63, 64]. Finally, water exchange times have been reported to be shorter at higher temperatures [40], and the difference of the water exchange time between *in vitro* and *in vivo* experiments may reflect the differences in mouse body temperature (36 °C) and room temperature (23 °C).

Our study has limitations. The relatively long echo time required to perform long Δ scans results in low SNR at high *b* values. Therefore, our method is limited to conditions where the DWI signal is quite high, such as in cerebral infarction, cancer, and white matter lesions. It is difficult to evaluate water permeability in the normal brain or in diseases such as Alzheimer's that are not associated with a high DWI signal. Other methods that utilize the kurtosis may be more useful for low SNR situations [65, 66]. In addition, the limitations previously reported for the exchange model also apply here [34]. That is, the model does not consider the possibility that T1 and T2 are different in intracellular and extracellular spaces, nor does it consider the anisotropy of water diffusion. Finally, observations were made at only one point 2 h after MCAO. Even though there was no substantial change in signal between the four sets of MbMtd DWI measurements taken for each mouse (a period of about 1.5 h, data not shown), the ischemic cascade is a dynamic process so a future longitudinal study of membrane permeability changes in MCAO model mice is important.

In conclusion, the present *in vivo* MbMtd DWI study found significant differences in the ADCs estimated across different *b* value ranges for animal models with and without aquaporin-4 expression in normal and ischemic tissue. Genotype-dependent differences in the ADC were particularly significant for the low *b* value range in normal tissue and for the high *b* value ranges in ischemic tissue. A significant difference in the ADCs measured at different diffusion-times was detected for both genotypes. Permeability may make a major contribution to the diffusion time dependence of the ADC, but the dependence is independent of genotype. Estimation of the parameters of a water exchange model quantified differences in water exchange-time that might be related to aquaporin-4 deficiency. The results suggest that MbMtd DWI might be useful for evaluating the efficacy of aquaporin-4 knockout targeted medicines and for the clinical diagnosis of aquaporin-4-related diseases.

## Supplementary Information

**Additional file 1: Supplementary figure. 1** The two-compartment model with inter-compartmental exchange. C_ex_(Td) and C_in_(Td) are the normalized extracellular and intracellular signals, respectively, at diffusion-time Td. t_ex_ and t_in_ are constants representing the inter-compartmental lifetimes. D_ex_ is the diffusion coefficient in the extracellular space. The diffusion coefficient in the intracellular space is modelled as “α/(Δ-δ/3)^β^”, where β is taken to be 1 in this work, and “α” is a fitting parameter with units of length squared if β = 1. The water signal from each compartment decreases with a rate constant equal to q^2^D, where the q-value is determined by the parameters of the motion probing gradient. **Supplementary figure. 2** The mean b-value dependent signal attenuation on each side of the brain. The data is the same as that shown in Figure 1 but plotted to highlight differences due to altered Δ. (a) Ipsilateral side of wild-type (WT) mice, (b) ipsilateral side of aquaporin-4 knockout (AQP4-KO) mice, (c) contralateral side of WT and (d) contralateral side of AQP4-KO mice. **Supplementary figure. 3** The b-value-dependent SNR on the ipsilateral (left) and contralateral (right) sides of the brain. The horizontal broken lines indicate SNR = 5. Data were excluded from the analysis if the SNR was less than 5. SNR: signal-to-noise ratio

## Data Availability

The datasets used and/or analyzed during the current study are available from the corresponding author on reasonable request.

## Abbreviations

ADC: Apparent diffusion coefficient
ANOVA: Analysis of variance
DWI: Diffusion-weighted imaging
IVIM: Intravoxel incoherent motion
MbMTd: Multi-*b*-value multi-diffusion-time
MCAO: Middle cerebral artery occlusion
MRI: Magnetic resonance imaging
ROI: Regions-of-interest
SNR: Signal-to-noise ratio
