# Supplementary material for: Exploring cell membrane water exchange in aquaporin-4-deficient ischemic mouse brain using diffusion-weighted MRI
Source: Eur Radiol Exp. 2021 Oct 7;5:44. doi: 10.1186/s41747-021-00244-y (PMC8494869; doi:10.1186/s41747-021-00244-y)
Supplement: Supplementary file 1 — Additional file 1: Supplementary figure. 1 The two-compartment model with inter-compartmental exchange. Cex(Td) and Cin(Td) are the normalized extracellular and intracellular signals, respectively, at diffusion-time Td. tex and tin are constants representing the inter-compartmental lifetimes. Dex is the diffusion coefficient in the extracellular space. The diffusion coefficient in the intracellular space is modelled as “α/(Δ-δ/3)β”, where β is taken to be 1 in this work, and “α” is a fitting parameter with units of length squared if β = 1. The water signal from each compartment decreases with a rate constant equal to q2D, where the q-value is determined by the parameters of the motion probing gradient. Supplementary figure. 2 The mean b-value dependent signal attenuation on each side of the brain. The data is the same as that shown in Figure 1 but plotted to highlight differences due to altered Δ. (a) Ipsilateral side of wild-type (WT) mice, (b) ipsilateral side of aquaporin-4 knockout (AQP4-KO) mice, (c) contralateral side of WT and (d) contralateral side of AQP4-KO mice. Supplementary figure. 3 The b-value-dependent SNR on the ipsilateral (left) and contralateral (right) sides of the brain. The horizontal broken lines indicate SNR = 5. Data were excluded from the analysis if the SNR was less than 5. SNR: signal-to-noise ratio [file 41747_2021_244_MOESM1_ESM.docx]

**ELECTRONIC SUPPLEMENTARY MATERIAL**


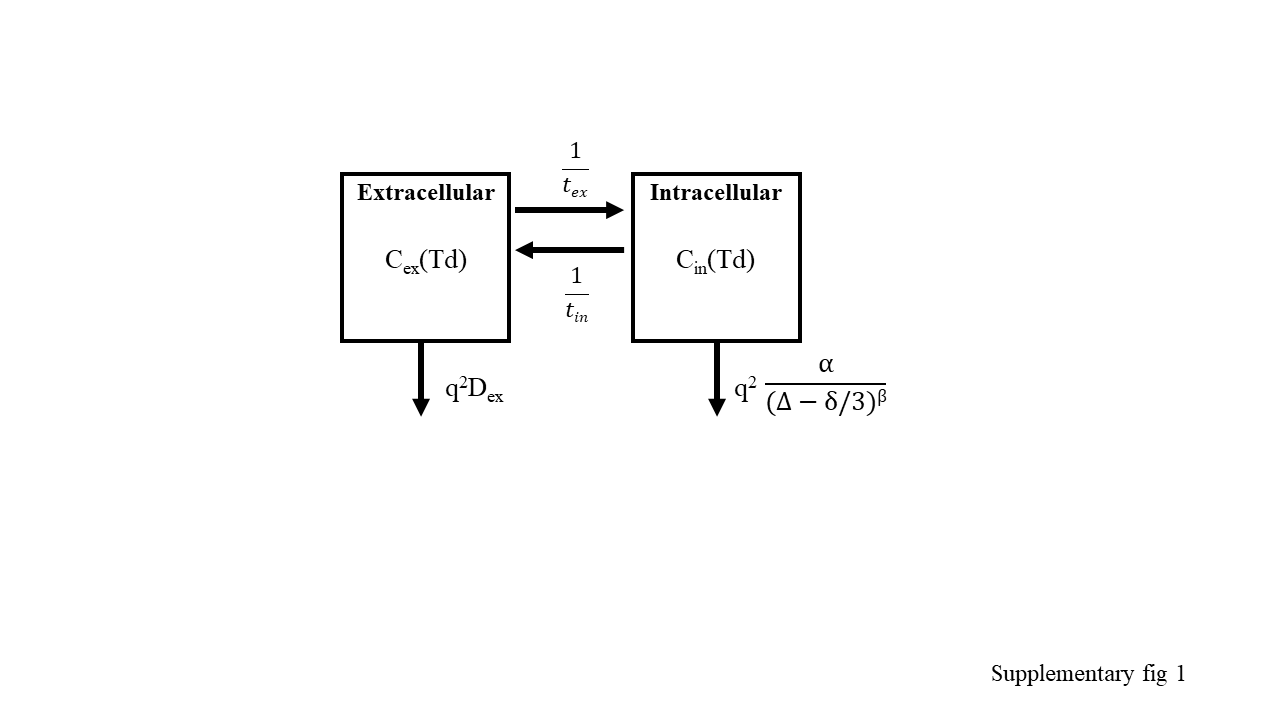


**Supplementary figure. 1**

The two-compartment model with inter-compartmental exchange. C_ex_(Td) and C_in_(Td) are the normalized extracellular and intracellular signals, respectively, at diffusion-time Td. t_ex_ and t_in_ are constants representing the inter-compartmental lifetimes. D_ex_ is the diffusion coefficient in the extracellular space. The diffusion coefficient in the intracellular space is modelled as “α/(Δ-δ/3)^β^”, where β is taken to be 1 in this work, and “α” is a fitting parameter with units of length squared if β = 1. The water signal from each compartment decreases with a rate constant equal to q^2^D, where the q-value is determined by the parameters of the motion probing gradient.


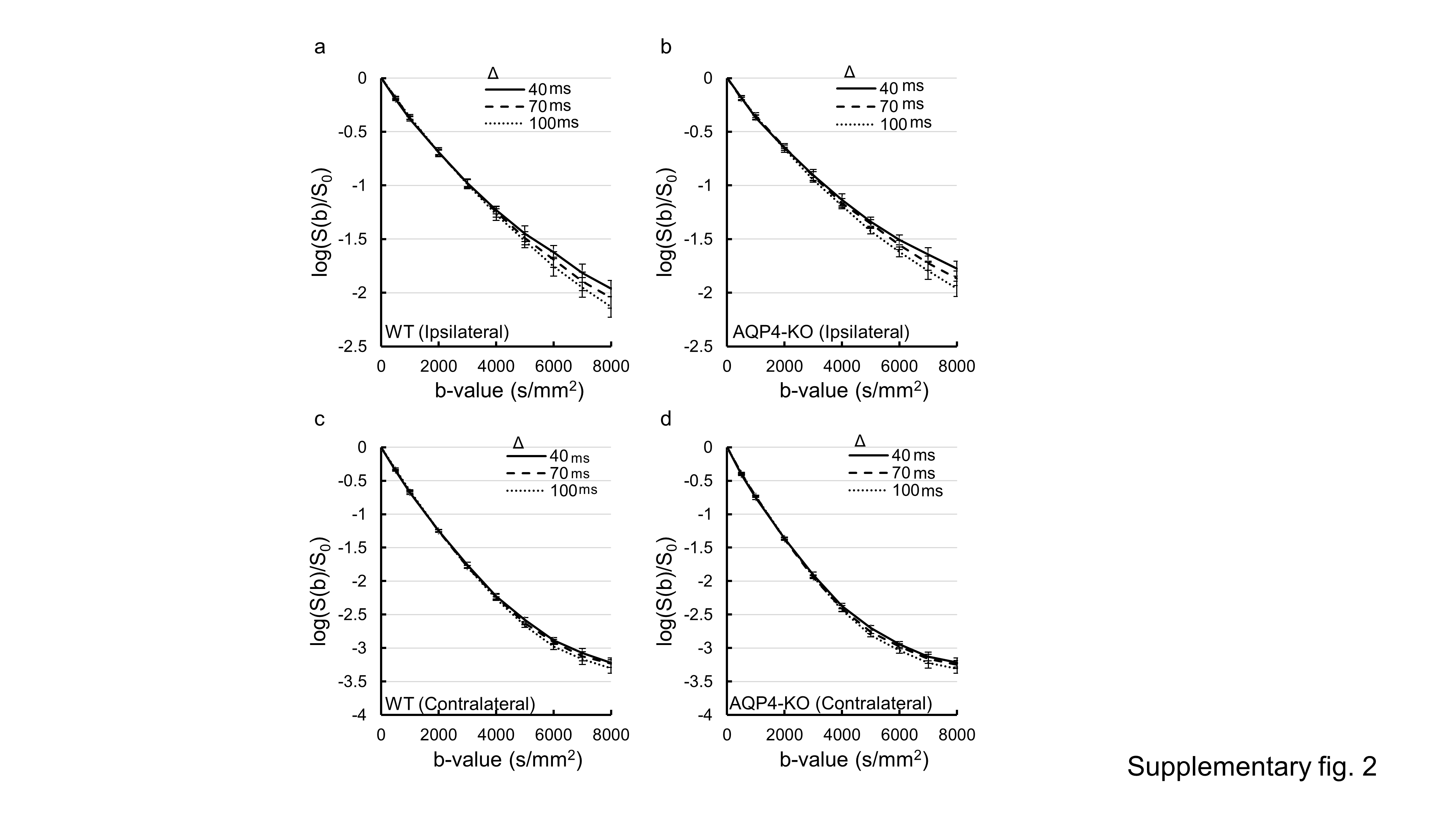


**Supplementary figure. 2**

The mean b-value dependent signal attenuation on each side of the brain. The data is the same as that shown in Figure 1 but plotted to highlight differences due to altered Δ. (a) Ipsilateral side of WT mice, (b) ipsilateral side of AQP4-KO mice, (c) contralateral side of WT and (d) contralateral side of AQP4-KO mice. AQP4-KO: aquaporin-4 knockout. WT: wild-type.


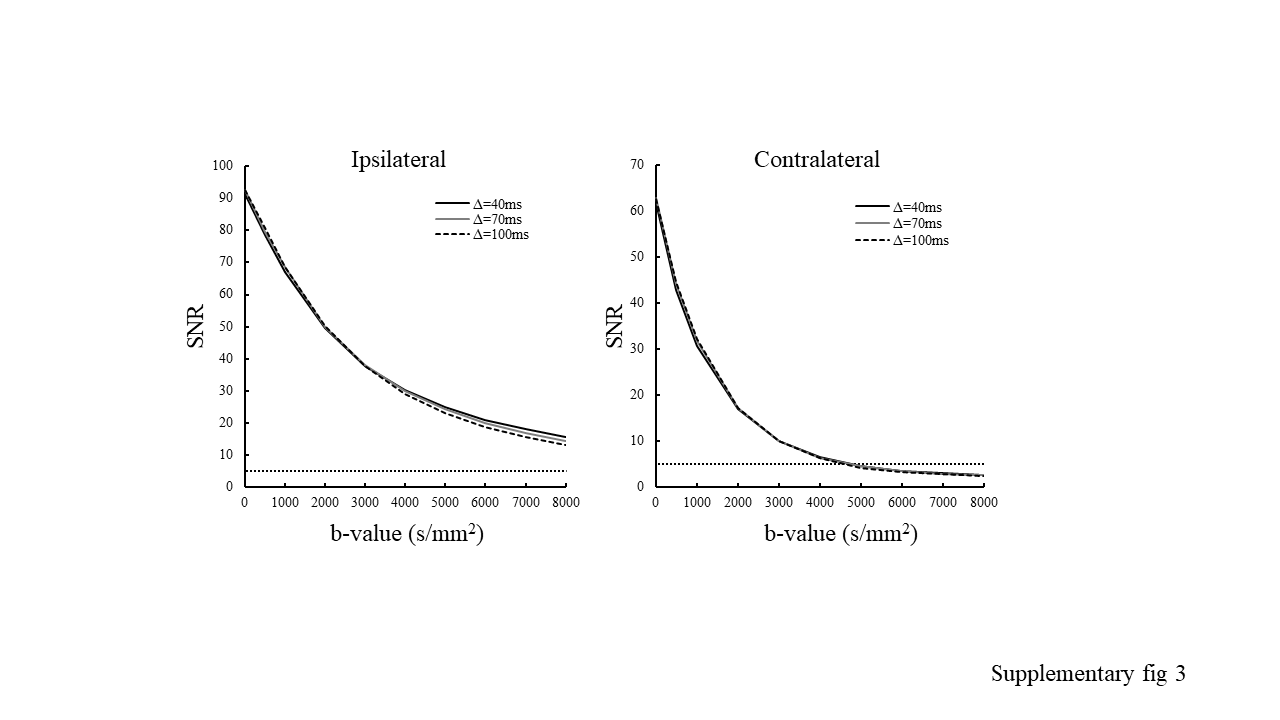


**Supplementary figure. 3**

The b-value-dependent SNR on the ipsilateral (left) and contralateral (right) sides of the brain. The horizontal broken lines indicate SNR=5. Data were excluded from the analysis if the SNR was less than 5. SNR: signal-to-noise ratio.
